# Soluble mediators of innate immunity in annelids and bivalve mollusks: A mini-review

**DOI:** 10.3389/fimmu.2022.1051155

**Published:** 2022-12-02

**Authors:** Laura Canesi, Manon Auguste, Teresa Balbi, Petra Prochazkova

**Affiliations:** ^1^ Department of Earth Environment & Life Sciences, University of Genoa, Genoa, Italy; ^2^ Laboratory of Cellular and Molecular Immunology, Institute of Microbiology of the Czech Academy of Sciences, Prague, Czechia

**Keywords:** Innate immunity, invertebrate, earthworm, bivalves, antimicrobial peptides, cytokines

## Abstract

Annelids and mollusks, both in the superphylum of Lophotrochozoa (Bilateria), are important ecological groups, widespread in soil, freshwater, estuarine, and marine ecosystems. Like all invertebrates, they lack adaptive immunity; however, they are endowed with an effective and complex innate immune system (humoral and cellular defenses) similar to vertebrates. The lack of acquired immunity and the capacity to form antibodies does not mean a lack of specificity: invertebrates have evolved genetic mechanisms capable of producing thousands of different proteins from a small number of genes, providing high variability and diversity of immune effector molecules just like their vertebrate counterparts. This diversity allows annelids and mollusks to recognize and eliminate a wide range of pathogens and respond to environmental stressors. Effector molecules can kill invading microbes, reduce their pathogenicity, or regulate the immune response at cellular and systemic levels. Annelids and mollusks are “typical” lophotrochozoan protostome since both groups include aquatic species with trochophore larvae, which unite both taxa in a common ancestry. Moreover, despite their extensive utilization in immunological research, no model systems are available as there are with other invertebrate groups, such as *Caenorhabditis elegans* or *Drosophila melanogaster*, and thus, their immune potential is largely unexplored. In this work, we focus on two classes of key soluble mediators of immunity, i.e., antimicrobial peptides (AMPs) and cytokines, in annelids and bivalves, which are the most studied mollusks. The mediators have been of interest from their first identification to recent advances in molecular studies that clarified their role in the immune response.

## Annelids

Annelids, or segmented worms, are invertebrates with a true coelom filled with coelomic fluid containing several coelomocyte populations. Annelids include polychaetes (marine worms), oligochaetes (earthworms), and leeches. All members of this group are, to some extent, segmented, i.e., they consist of segments that are made up of sub-segments partially intersecting with the body cavity. Since their body is open to the environment, the coelomic cavity is not aseptic, and therefore they had to evolve extremely effective weapons against unwanted enemies. Effector immunocytes called amoebocytes are involved in a broad range of defense functions, including phagocytosis modulated by humoral components, opsonins, which are complement-like molecules, and lectins. The coelomic fluid of annelids contains various antimicrobial factors like lysozyme, hemolytic factors that bind sphingomyelin (fetidin, lysenin, lysenin-related proteins), antimicrobial peptides (AMPs), proteases (lumbrokinase, catalase, superoxide dismutases), cytolytic proteins (coelomic cytolytic factor (CCF)), clotting and coagulation factors, and enzyme-activation-based cascades (prophenoloxidase cascade) (1). Bioactive agents in the coelomic fluid also include several metabolites, like drilodefensins, responsible for the inhibitory effects of polyphenols in the earthworm digestive tract (2), and metallothioneins, small metal-binding proteins responsible for the detoxification and regulation of heavy metals (3). Notably, the humoral defense also includes pattern recognition receptors (PRRs) (such as Toll-like receptors (TLRs) and CCF) that are frequently based on lectin-saccharide interactions designed to recognize highly conserved structures present in different microorganisms (pathogen-associated molecular patterns (PAMPs)). Consequently, cellular and humoral pathways of the innate defense are orchestrated by cytokine-like factors (4).

### AMPs

AMPs are evolutionarily ancient molecules produced by all living organisms, including annelids. They represent the first line of defense against microbes but also regulate commensal symbionts at various anatomical sites. Although more than 2,000 AMPs have been described in a wide range of organisms, the number of AMPs described in annelids is very small (**Table 1**).

**Table 1:** Antimicrobial peptides in annelids.

AMP family	Name	Structure	Annelida group	Species	Habitat	Ref.
**Lumbricins**	lumbricin-1 Hm-lumbicin PP-1 lumbricin-PG lumbricin, LuRP	proline-rich	oligochaetes leeches oligochaetes oligochaetes oligochaetes	*L. rubellus* *H. medicinalis* *P. tschiliensis* *P.guillelmi* *E. andrei*	terrestrial freshwater terrestrial terrestrial terrestrial	(5) (6–9)
**Macins**	theromacin Hm-neuromacin Hm-theromacin	α-helix/ β-sheet	leeches	*T. tessulatum* *H. medicinalis* *H. medicinalis*	freshwater	(6, 10)
**BRICHOS-AMPs**	arenicin-1 arenicin-2 arenicin-3 alvinellacin capitellacin nicomicin-1 nicomicin-2	β-sheet	polychaetes	A. marina *A. marina* *A. marina* *A. pompejana* *C. teleta* *N. minor* *N. minor*	marine	(11–15)
	hedistin	α-helix	polychaetes	*H. diversicolor*	marine	(16)
	perinerin	UN	polychaetes	*P. aibuhitensis*	marine	(17)
	Ms-hemerycin	α-helix	polychaetes	*M. sanguinea*	marine	(18)

A. marina, Arenicola marina; A. pompejana, Alvinella pompejana; C. teleta, Capitella teleta; E. andrei, Eisenia andrei; H. diversicolor, Hediste diversicolor; H. medicinalis, Hirudo medicinalis; L. rubellus, Lumbricus rubellus; LuRP, lumbricin-related peptide; M. sanguinea, Marphysa sanguinea; N. minor, Nicomache minor; P. aibuhitensis, Perinereis aibuhitensis; P. guillelmi, Pheretima guillelmi; P. tschiliensis, Pheretima tschiliensis; T. tessulatum, Theromyzon tessulatum; UN, unknown.

The first discovered AMP in annelids was lumbricin-1 from the earthworm *Lumbricus rubellus* (5). Related peptides were subsequently identified in several annelids: Hm-lumbricin from the leech *Hirudo medicinalis* (6), peptide PP-1 from the Asian earthworm *Metaphire tschiliensis* (7), lumbricin-PG from the earthworm *Pheretima guillelmi* (8), and lumbricin and lumbricin-related peptide from the earthworm *Eisenia andrei* (9). These AMPs are enriched with prolines and display a broad spectrum of antibacterial activities. However, they are not inordinately abundant and probably do not represent their primary biological function. Their mechanism of action on microorganisms remains unknown.

Macins are quite long cationic cysteine-rich AMPs with an α-helix/β-sheet structure. In leeches, the group includes theromacin from *Theromyzon tessulatum* (6) and Hm-neuromacin and Hm-theromacin from *H. medicinalis* (10). In addition to their antimicrobial activity, these AMPs are associated with regenerating damaged nerve cords. Their mechanism of action consists of the induction of bacteria aggregation followed by membrane permeabilization (19).

The BRICHOS-AMPs (Bri2 chondromodulin and prosurfactant protein C) are cysteine-rich AMPs only found in polychaetes. They arise from a precursor containing the BRICHOS domain, a hydrophobic region, and a cysteine-rich C-terminal region folding into a double-stranded β-sheet. Interestingly, the BRICHOS domain is linked to amyloid formations occurring in several major human diseases like Parkinson’s and Alzheimer’s (20). The first described members of the BRICHOS family were arenicin 1 and 2 in the polychaete *Arenicola marina* (11). The subsequently identified arenicin 3 differs in its primary sequence and contains an extra disulfide bond (12). A similar AMP, alvinellacin, was identified in the hydrothermal Pompeii worm *Alvinella pompejana*, a deep ocean polychaete associated with hydrothermal vents (13). By genome screening of *Capitella teleta*, another member of the BRICHOS-AMPs family called capitellacin was described (14). Nicomicin-1 and-2 were discovered in the artic polychaete *Nicomache minor*, which lives in frigid waters (15). Except for Nicomicin-2, all BRICHOS-AMPs exhibit a broad spectrum of antimicrobial activities against Gram-negative and Gram-positive bacteria, including multi-resistant species and fungi. These AMPs have membranolytic activity, causing membrane permeabilization of microbes within minutes. Arenicins are also hemolytic and cytotoxic to mammalian cells (21).

Hedistin, characterized by a linear α-helix structure, has only been described in the marine polychaete *Hediste diversicolor* (16). Hedistin contains bromotryptophan residues, making it stereochemically less susceptible to proteolysis. It is expressed by NK-like cells, suggesting its involvement in innate immune responses, which is confirmed by its comprehensive antimicrobial activity (16). Perinerin from the Asian marine clamworm *Perinereis aibuhitensis* is a hydrophobic and highly basic peptide with antimicrobial and bactericidal activity (17). It contains four cysteine residues forming disulfide bonds and acts through its pore-forming activity. Ms-hemerycin was identified in the marine lugworm *Marphysa sanguinea*, which lives in a swamp (18). This small peptide forms an unordered structure with a partial helical region. It has strong antimicrobial activity, but its mode of action is unconventional (**Table 1**).

### Cytokines

Most of our understanding of cytokines and their receptors in invertebrates relies on functional assays and similarities at the physicochemical level. The existence of cytokines in annelids, specifically IL-1-α, TNF-α) and POMC-derived peptide-like molecules, is indicated by work using immunohistochemical methods for their detection (22). Flow cytometry using monoclonal antibodies against human or mouse cytokines detected TNF-α and tumor growth factor-α (TGF-α) in earthworm coelomocytes (23).

Several genomic sequence analyses suggest that the innate immune system of invertebrates and vertebrates evolved independently and that invertebrate cytokine-like molecules and vertebrate cytokines do not have the same evolutionary ancestry. One of the molecules that can be considered a cytokine is coelomic cytolytic factor (CCF), which was first found in the earthworm *E. fetida* and later in other earthworm species (24, 25). CCF exists in both membrane and soluble forms and functions primarily as a PRR (4). However, some of its properties indicate that it also functions as a cytokine. Based on similar lectin-like activities, CCF shares functional analogies with mammalian tumor necrosis factor (TNF-α). They both lyse the TNF-sensitive tumor cell line L929, and in African and American trypanosomes, they are secreted after LPS stimulation, have opsonizing properties, bind antigens *via* lectin-like interactions, and increase membrane conductance in mammalian cells by interacting with ion-channels or ion-channel-coupled molecules. Despite the functional analogies of CCF and TNF-α, they do not display any gene or amino acid sequence homology, which indicates distinct evolutionary origins and convergence of invertebrate and vertebrate cytokine molecules (26–28) (**Table 2**).

**Table 2:** Cytokines in annelids and bivalve mollusks.

Cytokines		
Type	Annelids	Bivalve mollusks
TNF-α	**CCF**: found in earthworms; a functional analog of mammalian TNF-α (27)	**AbTNF-α**: homolog of TNF-α in abalone *Haliotis discus discus*; expressed in both immune and non-immune tissues; activation by pathogenic bacteria, viruses, LPS (29) **CgTNF-1, CgTNF-2** in *C. gigas* hemocytes, activated by bacterial stimuli (30)
IL-17	**IL-17**: 7 genes for IL-17 in polychaete *C. teleta*; function was not identified (31)	**IL-17**: 15 genes for IL-17 in *P. fucatamartensii (* **PmIL-17** *)*, 10 in *C. gigas (* **CgIL-17** *)*, 6 in *M. galloprovincialis*, 10 in scallop *Mizuhopecten yessoensis;* mucosal immunity; upregulation after bacterial stimulation; inflammatory response; NF-ĸB signaling pathway (32–35)
AIF-1	**AIF-1**: detected in leech *H. medicinalis*; chemo-attractant for macrophages that induces their migration towards an inflammation site (36)	**CgAIF-1**: identified in *C. gigas;* calcium-binding cytokine associated with immune cell activation and inflammatory response; activated by multiple PAMPS; enhances mRNA levels of MIF, TNF-α, and IL-17 (37)
EMAP-II	**EMAP-II**: chemokine for microglial cells, found in earthworms and leeches (3, 4)	n/i
MIF	n/i	**MgMIF**: identified in *M. galloprovincialis*; downregulated following a challenge by bacteria and fungi (38); **CfMIF**: identified in scallop *Chlamys farreri*; upregulated by LPS, PGN, and β-glucans; rCfMIF promotes migration of sheep fibroblast into scraped spaces *in vitro* (39) **PoMIF**: identified in oyster *Pinctada fucata*; upregulation upon bacterial stimulation (40)
IFN-γ	n/i	**CgIFNLP**: identified in oyster *C. gigas* has an IFN domain; shares low sequence identities with vertebrate IFNs, but displays a similar three-dimensional structure with class II helical cytokines; promotes the apoptosis and phagocytosis of hemocytes; responds to poly (I:C) stimulation; several components of the **IFN-like system**, activation by virus, poly (I:C), LPS; antiviral immunity (41–44)
Prokineticins	n/i	**Astakins:** homologs of vertebrate prokineticins; regulates hematopoiesis and the function of immune cells; lack of the essential sequence for proper signaling and binding of the G-protein coupled receptors; different receptor-mediated mechanisms; characterized in oyster *C. gigas* with a CgATP synthase β subunit protein involved in signaling in hemocytes (45)

AbTNF-α, tumor necrosis factor alpha homolog in Haliotis; AIF-1, allograft inflammatory factor- 1; CCF, coelomic cytolytic factor; CfMIF, macrophage migration inhibitory factor in Chlamys; C. gigas, Crassostrea gigas; CgAIF-1, allograft inflammatory factor-1 in Crassostrea; CgIFNLP, interferon gamma like protein in Crassostrea; CgIL-17, interleukin 17 in Crassostrea; CgTNF-1/2, tumor necrosis factor alpha in Crassostrea; C. teleta, Capitella teleta; EMAP-II, endothelial monocyte-activating polypeptide II; IL-17, interleukin 17; IFN-γ, interferon gamma; n/i, not identified; LPS, lipopolysaccharide; MgMIF, macrophage migration inhibitory factor in Mytilus; MIF, macrophage migration inhibitory factor; NF-ĸB, nuclear factor kappa B; PAMPs, pathogen-associated molecular patterns; PGN, peptidoglycan; P. fucata martensii, Pinctada fucata martensii; PmIL-17, interleukin 17 homolog in Pinctada; PoMIF, macrophage migration inhibitory factor in Pinctada; rCfMIF, recombinant CfMIF; TNF-α, tumor necrosis factor alpha.

Endothelial monocyte-activating polypeptide II (p43/EMAP-II), a chemokine, has been described in earthworms and leeches (46, 47). Endothelial monocyte-activating polypeptide II (p43/EMAP-II), a chemokine, has been described in earthworms and leeches (46, 47). In mammals, EMAP-II is released after processing the p43 precursor and functions as a pro-inflammatory cytokine chemotactic for monocytes and granulocytes and as a marker of microglial cell reactivity induced by brain tissue inflammation or neurodegeneration, but it has more pleiotropic biological activities (48). Similarly, the medicinal leech *Hm*EMAP-II is processed from the *Hm*p43 precursor, which is released by apoptotic cells and serves as a monocyte chemo-attractant (49). The *Hm*EMAP-II has a chemo-attractant effect on microglial cells, *e.g.*, the effect of mammalian EMAP-II on monocytes. Further, gene expression of HmEMAP-II, which can recruit phagocytic cells at lesion sites, is controlled by *Hm*TLR (46). In earthworms, greater expression of EMAP-II is accompanied by a greater abundance of mccEaTLR in seminal vesicles, suggesting its involvement in signal transduction (50) (**Table 2**).

Interleukin 17 (IL-17), the most conserved cytokine across animal phyla, is an essential factor in innate immunity. In humans, it is produced by activated T lymphocytes and cells participating in innate immunity, *e.g.*, mucosal epithelial cells (51). IL-17 has also been described in some annelids, including the segmented worm *C. teleta*, whose genome screening identified seven genes for IL-17. However, the function of IL-17 molecules has not been identified (31). Although the innate immunity signaling pathways involving cytokine action are not well described in annelids, several pathways have been identified by analysis of the coelomic fluid transcriptome of two earthworm species, *E. andrei* and *fetida.* In doing so, specific innate immune pathways were identified, such as the complement cascade, regulation of autophagy, NK-mediated cytotoxicity, chemokine-, MAPK-, mTOR-, NOD-like receptor-, and TLR- and Jak-STAT signaling (52). This suggests that many hidden molecules functioning as cytokines have not been discovered yet, and establishing their ability to build molecular networks is needed (**Table 2**).

Allograft inflammatory factor-1 (AIF-1) is a calcium-binding protein induced by cytokines participating in the allograft immune and inflammatory response of humans. It is expressed by monocytes/macrophages, the homolog of which was detected in the medicinal leech *H. medicinalis*, where it serves as a potent chemo-attractant for macrophages, and it induces their migration towards sites of inflammation (36) (**Table 2**).

## Bivalves

Among *Mollusca*, bivalves, including edible and aquacultured species (oysters, mussels, clams, etc.), are the most widely studied relative to their immune defenses. Bivalve hemocytes are responsible for cell-mediated immunity through the combined action of phagocytic processes and humoral defense factors such as agglutinins (*e.g.*, lectins), lysosomal enzymes (*e.g.*, acid phosphatase, lysozyme), reactive oxygen intermediates, and cytokines and various AMPs (53). Bivalve hemolymph serum contains a wide range of secreted components that participate in agglutination, opsonization, degradation, and encapsulation of microorganisms, as well as clotting and wound healing. In general, non-self recognition (in the form of PAMP) by soluble lectins and other PRRs and opsonins in hemolymph and hemocytes, which are present in the circulation and tissues, triggers signaling transduction cascades that release several effectors from tissues and hemocytes that lead to humoral and cellular immune responses, depending on the nature and location of the immune stimuli. An overview of the most recent accomplishments in the fields of recognition, agglutination, and opsonization (lectins, fibrinogen-related domain-containing proteins, C1q domain-containing proteins, lysozymes, bactericidal/permeability-increasing proteins, and other pore-forming molecules, proteases, and protease inhibitors, cathepsins, phenoloxidase cascade) is provided by Gerdol et al. (54).

### AMPs

From the first identification (20 years ago), in the hemolymph of the mussel *Mytilus galloprovincialis*, of a peptide sharing sequence similarity with the arthropod defensin, and therefore named *Mytilus galloprovincialis* Defensin-1 (MGD-1) (55), research on bivalve AMPs has steadily increased. With advances in molecular technologies, different AMPs have been identified in several bivalve species (mussels, oysters, clams, scallops), which are generally characterized by structural differences, different activities, differential tissue/cell expression, accessory functions, and often species-specific biological properties (reviewed in (54, 56, 57).

Resilience to pollution, pathogens, and changing environmental conditions means that some bivalve species are important models for studying the role of AMPs in adaptation and immunity. The Mediterranean mussel, *M. galloprovincialis*, which is not subjected to the same massive pathogen-associated mortalities as other bivalves, represents a unique model for studying AMPs. In this species, a remarkable abundance of AMPs has been reported, with antimicrobial activity against various bivalve and vertebrate pathogens, with different AMPs (including defensins, mytilins, and myticins) sharing antibacterial and antifungal properties; mytimycin appears to be a strictly antifungal protein. *Mytilus* AMPs are highly polymorphic and have high genetic variability: the recent assembly of the *M. galloprovincialis* genome showed that, although dozens of different sequence variants were identified for each AMP family, each individual possessed a unique combination of a small number of variants (58).

Myticin C (MytC), the most expressed AMP in adult mussels, has the highest RNA polymorphism relative to other mussel AMPs (59). MytC has been shown to inhibit the replication of bacteria, fish viruses, and human herpesvirus (56) and to have chemotactic and wound healing properties (59, 60). In oyster hemocytes, treatment with mussel hemolymph, or synthetic MytC peptides, inhibits replication of OsHV-1. In addition, *in vivo* studies suggested that overexpression of MytC in hemocytes could alter the transcription of other immune-related genes (AMPs, complement proteins, lysozyme). These data suggested that MytC may also play a role as an immune system modulator with chemokine-like activities (56, 59). In *M. galloprovincialis*, the extensive repertoire of AMPs, endowed with a broad spectrum of immune functions, might have significantly contributed to the evolutionary advantage of mussels in adapting to extremely changing environments (56, 58).

Among AMPs, Big defensins (BD), the ancestors of β-Defensins, essential components of innate immunity in vertebrates and invertebrates, are a large family of AMPs characterized by a highly hydrophobic globular N-terminal domain, which is present in different phylogenetically distant species, including both marine and freshwater bivalves (61). Bivalves often display an expanded repertoire of BD sequences and thus represent an excellent case for investigating the processes behind the remarkable diversity of the primary sequence, both between and within species. In contrast with human β-defensins, BDs are characterized by antimicrobial activities over a wide range of osmolarity. Studies in bivalves showed that the presence of the conserved hydrophobic domain confers bactericidal activity even at high salt concentrations (62). Moreover, bivalve BD displays a characteristic mechanism of action, i.e., the N-terminal domain drives bacteria-triggered peptide assembly into antimicrobial aggregates termed “nanonets,” which entrap microbes and prevent invasive pathogens from entering host cells (63). This effect has been described for the Cg-BigDef1 in *C. gigas* (62) and the ApBD1 in the scallop *Argopecten purpuratus* (64).

An exhaustive list of AMPs isolated from marine bivalves and their main characteristics (peptide sequence, length, net charge, percent hydrophobic residues, structure, antimicrobial activity), updated to 2017, is provided in an article by Zannella C, et al. Microbial diseases of bivalve mollusks: infections, immunology, and antimicrobial defense (57). As mentioned above, most data refer to *Mytilus* sp. and *Crassostrea gigas* (57). More recently, other AMPs have also been characterized, *i.e.*, Myticusin-beta in *Mytilus coruscus* (65), Myticalin in *M. galloprovincialis* (66), and hemoglobin-derived polypeptides in *Tegillarca granosa* (67).

### Cytokines

Recent developments in the study of bivalves are based on studies first carried out on Pacific oyster *C. gigas*, one of the most widely cultivated marine species. Partly because of its commercial importance, Guofan Z. et al., using genetic and genomic techniques, sequenced the genome in 2012 (68). The sequencing led to a significant increase in studies on bivalve cytokines (roughly 70% of all publications on the subject have occurred in the last decade, according to PubMed).

Pioneering work in the early ‘90s shed the first light on the conservation of cytokine-like molecules in mollusks (69, 70). Subsequent studies indicated that host defense mechanisms in different invertebrate groups, including bivalves, can be modulated by a cytokine-like network like that in vertebrates (71–73). However, until the identification of the first molecules with a cytokine-like activity (24, 74), most information relied on functional assays, using heterologous cytokines and antibodies directed towards vertebrate cytokines and their receptors.

The first studies were limited to evolutionarily conserved factors identifiable by sequence similarities, such as IL-17 (32), MIF (38), AIF-1 (37), and TNF-α (29, 30). The functional role of these conserved cytokines was indicated by their increased expression in response to bacterial stimuli. Most current information for IL-17 comes from phylogenetic studies, identification of related signaling pathways, and effector mechanisms in models of *M. galloprovincialis* and *C. gigas* (33–35). IL-17 plays a crucial role in mucosal immunity in mussels, just like in vertebrates (35), which was first identified in *C. gigas* (32) and later on in *Pinctada fucata* (75). Further genomic searching found that there were fifteen IL-17 genes in *P. fucata martensii*, ten in *C. gigas*, six in *M. galloprovincialis*, and ten in scallop *Mizuhopecten yessoensis* (31) (**Table 2**). At least some of these IL-17 molecules, *e.g.*, PmIL-17-2 from *P. fucata martensii and CgIL-17-5* from *C. gigas*, were found to be involved in the innate immune response after bacterial stimulation (31, 34). The inflammatory IL-17 cytokines are encoded by a diverse gene family leading to expanded IL-17 repertoires in various marine invertebrates. This can be seen, for example, in the study of 16 mussel genomes, which revealed 379 unique IL-17 sequences and 96 unique IL-17 receptor variants (35). Phylogenetic analysis revealed that all detected invertebrate IL-17 genes, from both annelids and mollusks, are clustered into one group, suggesting a common ancestral gene of these invertebrate IL-17s (31) (**Table 2**).

However, for many other divergent cytokines, their presence and function in bivalves remained elusive and can only be demonstrated indirectly; interferon-γ is a good example. In *M. galloprovincialis* hemocytes, human recombinant IFN-γ was shown to activate members of a STAT-like pathway; moreover, hemocyte pretreatment with IFN-γ increased bacteria-induced STAT1-like phosphorylation, indicating that the function of mussel hemocytes may be physiologically regulated *in vivo* by endogenous IFN-γ-like cytokines (76). Since the release of the genome of *C. gigas* and other species, several components of the IFN-like system (IFN-like molecules, IFN-like receptors, and components of related signaling pathways) have been identified (41–43). Interestingly, the IFN system of bivalves can be activated by virus or poly (I:C) challenges and further regulate the antiviral response of hemocytes; however, some components also showed a positive response to other immunostimulants, such as lipopolysaccharide (LPS) and bacteria. Unlike vertebrate IFN systems, the bivalve IFN-like system may participate in multiple biological activities, such as antibacterial immunity (reviewed in (44) (**Table 2**).

Other cytokines that developed independently in different invertebrate groups are now being identified. For example, Astakines, considered homologous of vertebrate prokineticins, regulate hematopoiesis and immune cell function. Astakines lack the AVITGA sequence in the N-terminus essential for proper signaling and binding of G-protein coupled receptors, indicating the presence of distinct receptor-mediated mechanisms in invertebrates. Recently, a *C. gigas* astakine was identified and characterized using a CgATP synthase β subunit protein, which is involved in hemocyte signaling (45).

Whatever the role of the various bivalve cytokines, transcriptomic data indicate modulation within different species in response to environmental stressors ( (77) and refs. therein). However, little is known about how cytokine expression is physiologically regulated. Recent studies underlined the role of different miRNAs in the expression of TNF-α and IL-17 in oyster hemocytes (78, 79).

## Conclusions and perspectives

Annelids and bivalve mollusks represent important invertebrate groups for investigating innate immunity, particularly for the wide range of soluble immune mediators, which show a large diversity in structure and functions.

With regards to AMPs in annelids, three main classes have been identified so far in oligochaetes (lumbricins), polychaetes (BRICHOS-AMPs), and leeches (macines). Bivalves, in particular marine species, display a much wider repertoire of different AMPs (55), with some of them (*i.e.*, myticins, big defensins) also showing immune-related functions other than direct antibacterial activity.

Several soluble factors endowed with cytokine-like activity have been described in annelids and bivalves; however, few are structurally conserved in both groups relative to their vertebrate counterparts (*i.e.*, IL-17), which underlies their distinct evolutionary origin. From this perspective, research on invertebrate cytokines still represents an open and promising field for the identification of new immunoactive molecules.

Moreover, the properties of AMPs and cytokines in both groups of invertebrates suggest they have application potential in human health and the environment, especially given the global problem of antibiotic resistance. The increasing appearance of bacterial strains resistant to antibiotics has promoted an intense search for new anti-infective drugs that can be an effective alternative to conventional antibiotics. Invertebrate AMPs are in the spotlight as innovative drug candidates not only for the treatment of infectious diseases but also for their immunomodulatory potential.

To develop AMPs as efficient and safe new drugs, their mode of action needs to be fully understood. Despite their advantages, selecting suitable AMPs for clinical use is progressing very slowly. Pharmaceutical companies have had little interest in developing new antibiotics over the last 30 years because of high production costs and short efficacy periods (since bacterial resistance to new drugs develops rapidly). Although microorganisms can develop resistance to AMPs as a consequence of continuous selection pressure, AMPs still present several important advantages over antibiotics, e.g., they are much less stable in the environment and thus much reduced environmental impact. It was shown that cationic AMPs do not induce bacterial stress pathways and thus do not increase bacterial mutagenesis, which is not the case with antibiotics (80). Further, AMPs often use different mechanisms of action, including disruption of bacterial membranes, inhibition of metabolic pathways, and recruitment and activation of various immune cells (81).

Few annelid AMPs are currently being evaluated in clinical trials as treatments against various bacterial and fungal infections. An analog of Arenicin-3 was selected for preclinical trial by Adenium Biotech Ltd. for its effective action in treating urinary tract infections (82), but the trial was unfortunately stopped due to the company’s bankruptcy. The only molecule derived from the earthworm *L. rubellus* and successfully used in therapy, although not an AMP, is the enzyme lumbrokinase, used for its fibrinolytic properties to treat thrombus-related diseases. Similarly, since leech saliva contains large amounts of various enzymes with thrombolytic, anticoagulant, anti-inflammatory, and analgetic activity, leeches have been used to treat various diseases such as vascular disorders, cardiovascular disease, skin disorders, diabetic foot ulcers, migraine, knee osteoarthritis, and even in cosmetic surgery (83). The best-known enzyme from leeches is hirudin, which was discovered in 1884 when an extract from *H. medicinalis* was found to have anticoagulant properties (84). Recently, hirudin derivatives (*e.g.*, lepirudin, desirudin) and hirudin analogs (*e.g.*, bivalirudin) have been used clinically as direct bivalent thrombin inhibitors. However, despite its effectiveness, safety, and few complications, leech treatment is still controversial.

Despite the enormous potential of marine biodiversity for discovering AMPs, no bivalve AMPs have been tested in clinical trials. If, on the one hand, their resistance to high salt concentrations may represent potential advantages in medical applications (57), on the other hand, development has been hampered by many issues, such as discrepancies between *in vitro* and *in vivo* tests and susceptibility to proteases and pH, which affects AMP half-life *in vivo* (85).

Annelids and bivalves represent a vast source of AMPs that could serve as potential candidates for antibacterial drug development in human and veterinary medicine. Due to their broad spectrum of antimicrobial activities and emerging new immune modulation functions, AMPs have great potential for biotechnological applications and continue to be the subject of many molecular and functional studies. Antimicrobial peptides (AMPs) have been successfully applied in various areas of human health, including clinical medicine and drugs. However, as with AMPs from humans and other sources, high cost, poor stability, and toxicity are disadvantages that limit the clinical development of AMPs (86). In this light, a promising biotechnology field is genetically engineered microorganisms, where natural AMPs can be expressed or modified to create improved antibacterial abilities and increased stability while reducing toxicity (86).

The same applies to their cytokines, which act as natural immunomodulators with potential new biomedical applications. However, our understanding of invertebrate cytokines is still limited compared to AMPs and hampers the development of biotechnological products. The multiple and complex immunoregulatory roles of annelid and bivalve cytokines represent a still largely unexplored field of research with substantial innovative potential. This particularly applies to those cytokines that are not structurally conserved and whose role and molecular machinery are still elusive. The application of multiple molecular and functional approaches will help to better define their biological role in immune homeostasis in health and disease.

## Author contributions

PP, MA, TB, and LC contributed to the writing of the manuscript. PP and LC provided critical review and input. All authors read and approved the final manuscript.

## Funding

This project has received funding from the European Union’s Horizon 2020 research and innovation program under the Marie Skłodowska-Curie grant agreement No 671881.

## Conflict of interest

The authors declare that the research was conducted in the absence of any commercial or financial relationships that could be construed as a potential conflict of interest.

## Publisher’s note

All claims expressed in this article are solely those of the authors and do not necessarily represent those of their affiliated organizations, or those of the publisher, the editors and the reviewers. Any product that may be evaluated in this article, or claim that may be made by its manufacturer, is not guaranteed or endorsed by the publisher.
